# Drain fluid iodine as a biomarker of anastomotic leak after low anterior resection in patients undergoing Gastrografin rectal tube flushes and omission of a diverting ileostomy: The GUSH study

**DOI:** 10.1111/codi.70031

**Published:** 2025-02-19

**Authors:** David A. Clark, Karen Dobeli, Darren Allen, Brett McWhinney, Michael Lonne, GUSH Study Collaborators, Carina Chow, Craig Hacking, Craig Harris, Jennifer Liang, Amanda Liesegang, John Lumley, Damien Petersen, Danielle Siganto, Andrew Stevenson, Jacobus Ungerer, Aleksandra Edmundson

**Affiliations:** ^1^ Department of General Surgery Royal Brisbane and Women's Hospital Brisbane Queensland Australia; ^2^ Mayne Academy of Surgery University of Queensland Brisbane Australia; ^3^ Department of Surgery St Vincent's Private Hospital Northside Brisbane Queensland Australia; ^4^ Faculty of Medicine and Health University of Sydney, and Surgical Outcomes Research Centre (SOuRCe) Sydney New South Wales Australia; ^5^ Department of Radiology Royal Brisbane and Women's Hospital Brisbane Queensland Australia; ^6^ Department of Chemical Pathology, Pathology Queensland Royal Brisbane and Women's Hospital Brisbane Queensland Australia

**Keywords:** anastomotic leak, biomarker, drain fluid, dual‐energy CT, Gastrografin, inductively coupled mass spectroscopy, iodine, spectral CT

## Abstract

**Aim:**

Anastomotic leak (AL) is the anathema of colorectal surgery and its occurrence constitutes a serious risk to patients and places a substantial burden on the health system. The analysis of extravasated intraluminal substances in drain fluid has shown promise for the early detection of AL. The aim of this study is to assess the measurement of drain fluid iodine as a biomarker of AL.

**Method:**

This prospective, observational, 2b exploration cohort study measured the iodine in drain fluid of patients undergoing a low colorectal anastomosis and without a diverting ileostomy (DI) when the rectal tube was flushed with Gastrografin®. Iodine was measured by dual‐energy computed tomography (DECT) and inductively coupled plasma mass spectroscopy (ICPMS).

**Results:**

Sixty‐six patients underwent a rectal resection and low colorectal anastomosis. Five patients experienced an AL. Four had grade C AL and returned to the operating theatre for peritoneal lavage and DI. The fifth was diagnosed at 30 days postoperatively and underwent image‐guided drainage (grade B). The mean drain fluid iodine was significantly elevated in patients who experienced an AL compared with those who did not, as measured by DECT and ICPMS. The mean iodine value was 6.05 mg/mL vs. 0.088 mg/mL (*p* < 0.0001) for DECT and 41 437 μmol/L vs. 3.81 μmol/L (*p* < 0.0001) for ICPMS.

**Conclusion:**

This study showed that drain iodine can be used as a sensitive indicator of early AL in patients undergoing a rectal resection with an extraperitoneal colorectal anastomosis and omission of a DI and when the rectal tube is flushed with Gastrografin following surgery.

What does this paper add to the literature?

This paper further evaluates drain fluid iodine as a novel drain fluid biomarker of anastomotic leak (AL) in patients administered Gastrografin rectal tube flushes. Few studies have investigated the extravasation of intraluminal substances. Intuitively, these are more likely to lead to the diagnosis of AL versus those that simply predict the possibility of a leak, such as inflammatory biomarkers.

## INTRODUCTION

Anastomotic leakage (AL) is the anathema of colorectal surgery, and its occurrence constitutes a serious risk to colorectal patients and places a substantial burden on the health system [1]. The risk of AL increases the closer an anastomosis is to the anal verge, and diverting ileostomy (DI) has been employed after low anterior resection to reduce the septic consequences of AL [2]. DI itself has risk, and readmission with dehydration and obstruction is a common event [3]. An alternative strategy to mandatory DI is to identify patients experiencing AL at an early stage, before the onset of sepsis, and immediately return them to theatre for lavage and diversion [4, 5]. If a DI is to be avoided, a mechanism for early detection of AL is imperative [5].

Systemic biomarkers (BMs) are nonspecific, have poor positive predictive value and may also measure the idiosyncratic reparative processes and pulmonary or wound complications [1, 6]. Drain fluid BM studies have shown promise in terms of early detection, with some studies showing an elevation in BM on the day prior to the onset of clinical signs of sepsis [7, 8]. Studies of extravasated intraluminal substances (EILS) have correlated well with AL and intuitively replicate the process of anastomotic leakage [8, 9]. These substances must be present in high quantities in the intestinal lumen and not in the peritoneal cavity. Amylase and iodine have been investigated [10, 11].

Gastrografin® (Bayer, Australia; GG) is a water‐soluble iodinated contrast solution commonly used for abdominal CT. It can be employed safely as an enema and is the radiological contrast used in the detection of AL. GG has high levels of iodine bound as diatrizoate and can be measured quantitatively through dual‐energy computed tomography (DECT) and inductively coupled plasma mass spectroscopy (ICPMS) [11, 12].

Rectal tubes are used to reduce the clinical consequences of AL and are usually flushed with saline to prevent blockage [13, 14]. They can be flushed with GG to maintain patency and deliver high levels of measurable iodine to the intestinal lumen. A pelvic drain placed adjacent to the anastomosis would allow sampling of the peritoneal fluid in the fixed pelvic cavity [11].

This 2b exploration (Phase 2) study aimed to assess the safety and efficacy of GG rectal tube flushes and the feasibility of detecting the iodine in GG in drain fluid samples by DECT and ICPMS as a BM of AL.

## METHOD

This prospective, observational, 2b exploration cohort study aimed to establish the safety and efficacy of the measurement of the iodine in drain fluid and stool by DECT and ICPMS when the rectal tubes are flushed with GG and a diverting ileostomy is omitted.

Informed consent was obtained from all participants and retained as per archiving policy. All complications were recorded and graded using the Clavien–Dindo classification [15]. Adverse events were notified to the relevant ethics committee. The study was performed at three tertiary hospitals in Brisbane, Australia.

Human research ethics approval was granted by the Royal Brisbane and Women's Hospital Human Research and Ethics Committee (HREC/2020/QRBW/64118), the Wesley Hospital Human Research and Ethics Committee (2020.17.327) and the St Vincent's Health and Aged Care Human Research Ethics Committee (HREC 20/10) for the multicentre study.

The trial was registered with the Australian New Zealand Clinical Trials Registry (ACTRN12620000710921).

### Participants

Patients with the following inclusion and exclusion criteria were invited to participate. Only patients who provided informed written consent were included.

Inclusion criteria were: (1) patients undergoing a rectal resection with an extraperitoneal anastomosis (i.e. within 10 cm of the anal verge), and without a diverting loop ileostomy; (2) placement of a pelvic drain and rectal tube at surgery. The only exclusion criterion was an allergy to iodine or GG.

A sample size of 60 was chosen and an AL rate of 8% was expected based on prior studies from the participating institutions [4, 9]. Participants were allocated into the AL group after AL was confirmed by imaging and clinical examination and following the diagnostic criteria set out by the International Study Group of Rectal Cancer [16].

### Administration of GG to the rectal tube

All patients had their rectal tube flushed with 30 mL of 0.25% (vol/vol) GG in saline, 6‐hourly [12]. The first morning flush was administered at 06:00. Samples of pelvic drain fluid were collected twice daily, morning (am) and afternoon (pm), while the drain was in situ. Samples were collected 30 min after flushing the rectal tube with GG. Stool specimens were collected from the rectal tube daily for the first 2 days half an hour after flushing with GG. The stool was collected as an internal control to ensure that the intraluminal iodine levels were high compared with the peritoneal fluid. All samples were deidentified at the time of collection and stored at room temperature until ICPMS and DECT measurement.

### Gastrografin safety and applications

Gastrografin® (diatrizoate, also known as amidotrizoate meglumine and sodium amidotrizoate, Bayer Australia; registered trademark of the Bayer group, Germany) is a hyperosmolar water‐soluble iodinated radiological contrast medium. GG is recorded as ARTG ID:10684 on the Australian Register of Therapeutic Goods maintained by the Therapeutic Goods Administration [11].

GG may be administered orally or as an enema and is used routinely in clinical practice. Its safety has been established in a number of randomized trials [17]. A meta‐analysis of contrast enemas found that procedures involving GG were safe with only one reported complication in the 1169 procedures studied. GG and Urografin were the most common agents used in these radiological procedures [18].

Apart from its routine use in radiology, GG has been safely employed orally in patients with small bowel obstruction and assessed as a prokinetic in prolonged postoperative ileus [19, 20].

### Iodine measurement by DECT

The application of DECT scanning in the measurement of iodine in clinical specimens was established in a prior study from this institution [11]. DECT, also known as spectral CT, is a new technology that allows simultaneous or near‐simultaneous acquisition of two datasets from the same anatomical region at different x‐ray tube voltages [21]. DECT is capable of differentiating materials with different atomic numbers and the organically bound iodine in GG is able to be simply quantified using a DECT protocol described in the literature and in Supplement 1 [12].

All measurements were performed by the same radiographer (KD) using the same DECT scanner for all drain fluid samples. The same three control samples were included in the scanned field with each CT acquisition.

### Iodine measurement by ICPMS

ICPMS is an analytical method that is able to measure trace levels of specific elements based on their molecular mass. The system heats the biological fluid to high levels, which breaks down chemical bonds. Temperatures can reach over 10 000 K (hotter than the surface of the sun). The resulting ions are then measured by mass spectrometry [22].

### Pelvic drains

Surgeons participating in this study routinely position a pelvic drain after a low anterior resection with an extraperitoneal anastomosis [2]. It is possible that drains may be malpositioned or dislodged after insertion and this may lead to a false negative result in the setting of AL detection. While it is not intended that all patients should undergo routine imaging to confirm drain position, the majority of those experiencing a clinical AL will undergo CT imaging as part of usual clinical practice, presenting an opportunity for the assessment of drain position in the pelvis.

### Rectal tubes

At the participating sites it is routine practice to insert a 28G Foley catheter anally and across the sphincter in all patients undergoing a rectal resection with an extraperitoneal anastomosis. Balloons are inflated with 12 mL of water or sutured in place with the balloon deflated. Rectal tubes are routinely flushed with 30 mL of saline four times a day by hand following surgery to prevent blockage. In this study, the saline flushes were replaced with 0.25% (vol/vol) GG in saline.

### Sample collection

Measurements of drain fluid iodine levels were made on days 1–5, or longer if the drain remained in situ after 5 days. Drain fluid samples were collected twice daily from Bellovac drains 30 min after administration of the morning or afternoon rectal tube flushes with 25% GG diluted in saline. Drain samples were deidentified and stored at room temperature until measurement with ICPMS and DECT.

A daily sample of rectal tube fluid was collected on the first two postoperative days to measure the intraluminal iodine level.

### Data collection

Eligible patients provided written consent for inclusion in the study. Patient demographics, surgery details and postsurgical information were collected after obtaining informed consent. All patient information was deidentified by the assignment of a unique numeric code known only to the investigators listed on this project.

Any adverse events observed were assessed for any temporal relationship to the administered GG flushes. AL is defined and graded by the criteria set out in the International Study Group in Rectal Cancer document [16].

### Blinding of samples for DECT and ICPMS

The drain fluid samples were blinded to the reporting radiologist and chemical scientist, and scanned as a batch to reduce bias. The results for the measured iodine in the drain fluid by DECT and ICPMS were not available to the treating surgeon or participants.

### Analysis and statistics

Data are presented either as mean ± standard deviation for continuous, normally distributed variables or as median and interquartile range for nonparametric data. Means were compared with the unpaired *t*‐test using GraphPad Prism 8 (GraphPad, San Diego, California, USA). Iodine levels in the drain fluid of patients who experienced an AL were compared with iodine levels of drain fluid of those who that did not experience an AL. All iodine measurements by DECT and ICPMS and statistical analysis were performed using deidentified data. Results are displayed graphically using GraphPad Prism 8. The *y*‐axis is presented as a logarithmic scale. A *p*‐value of <0.05 is considered statistically significant.

## RESULTS

Sixty‐six patients met the inclusion criteria and consented to the study. Five patients experienced an AL (7.6%) confirmed clinically and radiologically. Four of these were grade C Als, and patients were returned to the operating theatre and underwent laparoscopic peritoneal lavage and diverting ileostomy. The fifth AL was a grade B AL and was diagnosed and managed with image‐guided drainage at postoperative day 30 [16]. All five of the anastomotic leak group underwent surgery for a malignant indication.

**FIGURE 1:**
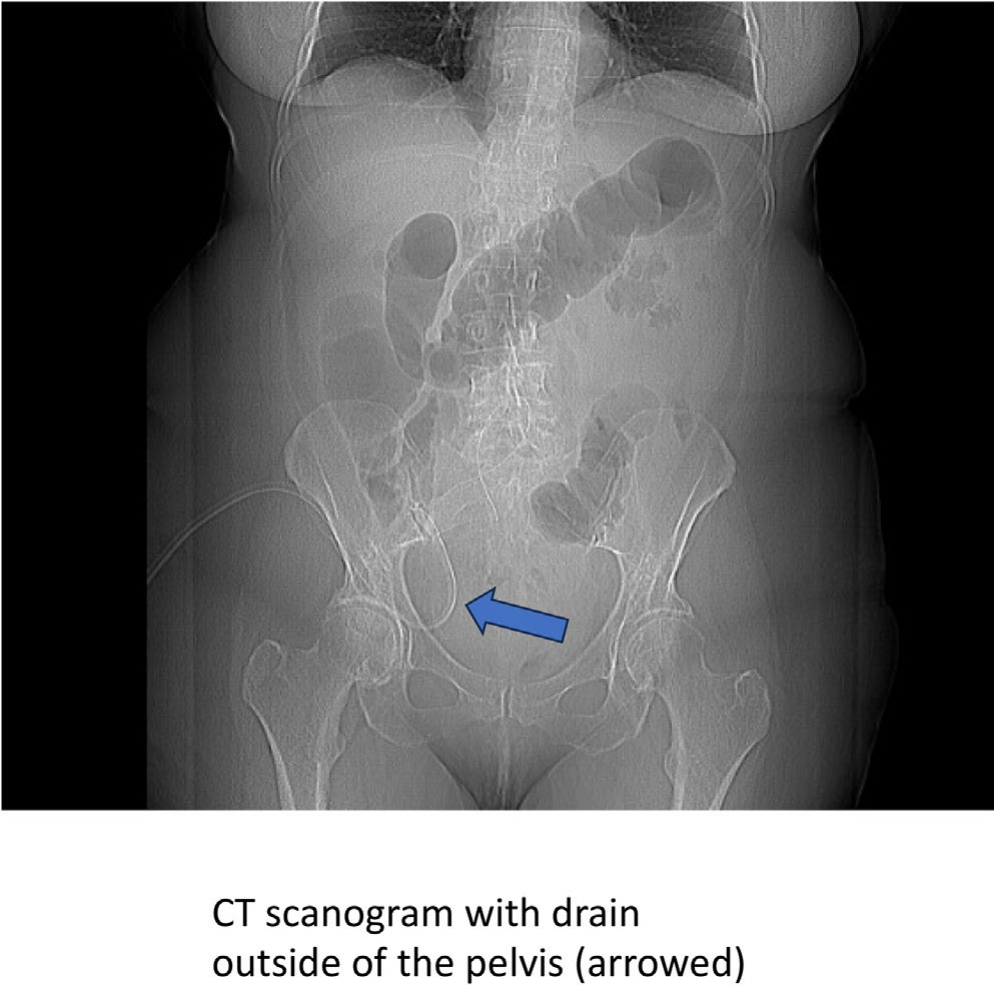
Imaging on day of anastomotic leak.

All four patients in the grade C AL group underwent a CT scan. In one of these patients the drain was not sitting in the pelvis (Figure 1). This was patient GUSH_65 and there was no elevation of drain fluid iodine seen on DECT or ICPMS.

The characteristics of the participants and operations are presented in Table 1. Those patients experiencing an AL were more likely to have a higher body mass index (BMI), a longer length of stay, a diagnosis of cancer, the pathology closer to the anal verge and a have a higher C‐reactive protein level at postoperative day 3. There were no adverse events related to GG flushing of rectal tubes nor was there any mortality in this cohort.

**TABLE 1:** Patient and operative characteristics.

Characteristic	Overall (*n* = 66)	AL (*n* = 5)	No AL (*n* = 61)	*p*‐value
	Mean (SD, %)	Mean (SD, %)	Mean (SD, %)	
Age (years)	60.3 (15.0)	61.4 (12.1)	60.2 (15.5)	0.868
Sex (count)				
Male	35 (53%)	3 (60%)	32 (52%)	
Female	31 (47%)	2 (40%)	29 (48%)	
Weight (kg)	85.0 (22.5)	101.1 (19.0)	83.6 (22.6)	0.099
Height (cm)	172.1 (9.6)	173.2 (10.6)	172.0 (9.7)	0.798
BMI (kg/m^2^)	28.6 (6.7)	34.3 (9.4)	28.1 (6.3)	**0.047**
Public/private (count)				
Public	16 (24%)	1 (20%)	15 (25%)	
Private	50 (76%)	4 (80%)	46 (75%)	
LOS (days)	6.3 (5.3)	22.2 (8.1)	5.05 (1.9)	**<0.0001**
Height of tumour above anal verge (cms)	11.0 (2.9)	7.4 (3.0)	11.3 (2.8)	**0.004**
Post‐operative day drain removal	4.2 (2.5)	13.0 (2.5)	3.6 (1.0)	**<0.0001**
Postoperative day for rectal tube removal	3.2 (0.9)	4.5 (1.3)	3.1 (0.8)	**0.002**
Postoperative day of full diet	1.7 (1.4)	3.6 (3.4)	1.6 (1.0)	**0.001**
Day 3 CRP	76.76 (73.5)	238.0 (121.2)	62.6 (49.1)	**<0.0001**
	Count (%)	Count (%)	Count (%)	
Operation (*n* = 66)				
Lap ULAR	20 (30%)	3 (60%)	17 (28%)	
Lap ULAR TaTME	1 (2%)	1 (20%)	0	
Robotic ULAR	7 (11%)	0	7 (11%)	
Robotic low anterior resection	12 (18%)	0	12 (20%)	
Lap low reversal Hartmann	3 (5%)	0	3 (5%)	
Lap low anterior resection	22 (33%)	0	22 (35%)	
Lap to open ULAR	1 (2%)	1 (20%)	0	
Stage (if cancer) (*n* = 51)				
Stage 1	18 (35%)	2 (40%)	16 (35%)	
Stage 2	17 (33%)	1 (20%)	16 (35%)	
Stage 3	13 (26%)	1 (20%)	12 (26%)	
Stage 4	3 (6%)	1 (20%)	2 (4%)	
TME grade if ULAR				
Grade 1	0	0	0	
Grade 2	1 (3.4%)	1 (20%)	0	
Grade 3	28 (96.6%)	4 (80%)	24 (100%)	
Anastomotic leak (%)		5 (8%)	0	
Return to OT (total)	6 (9%)	4 (80%)	2 (3%)	

Abbreviations: AL, anastomotic leak; CRP, C‐reactive protein; Lap, laparoscopic; LOS, length of stay; OT, operating theatre; TaTME, transanal TME; TME, total mesorectal resection; ULAR, ultralow anterior resection (TME).

Statistically significant *p* values in bold.

**FIGURE 2:**
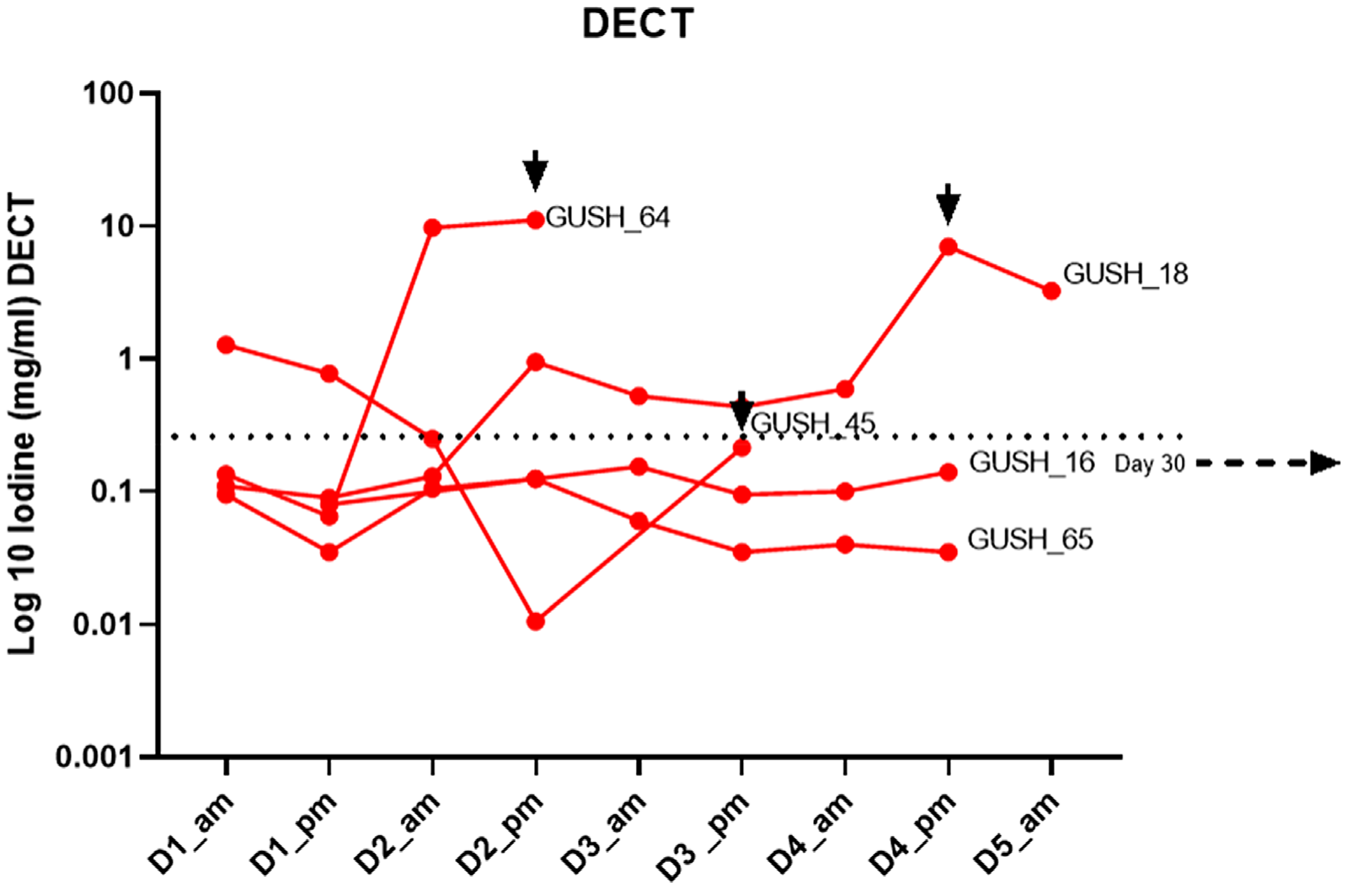
Time course of drain fluid iodine in patients experiencing anastomotic leak (AL) as measured by dual‐energy CT (DECT) (D1 etc., day 1 etc.). Arrows indicate the day of clinical diagnosis of AL; the dotted line represents the derived cutoff of the highest mean plus three standard deviations in the non‐AL group. The cutoff was 0.240 mg/mL in non‐AL drain fluid, seen as highest on D1. There was insufficient drain fluid collected for analysis for GUSH_65 on day 5, which was the day of AL.

**FIGURE 3:**
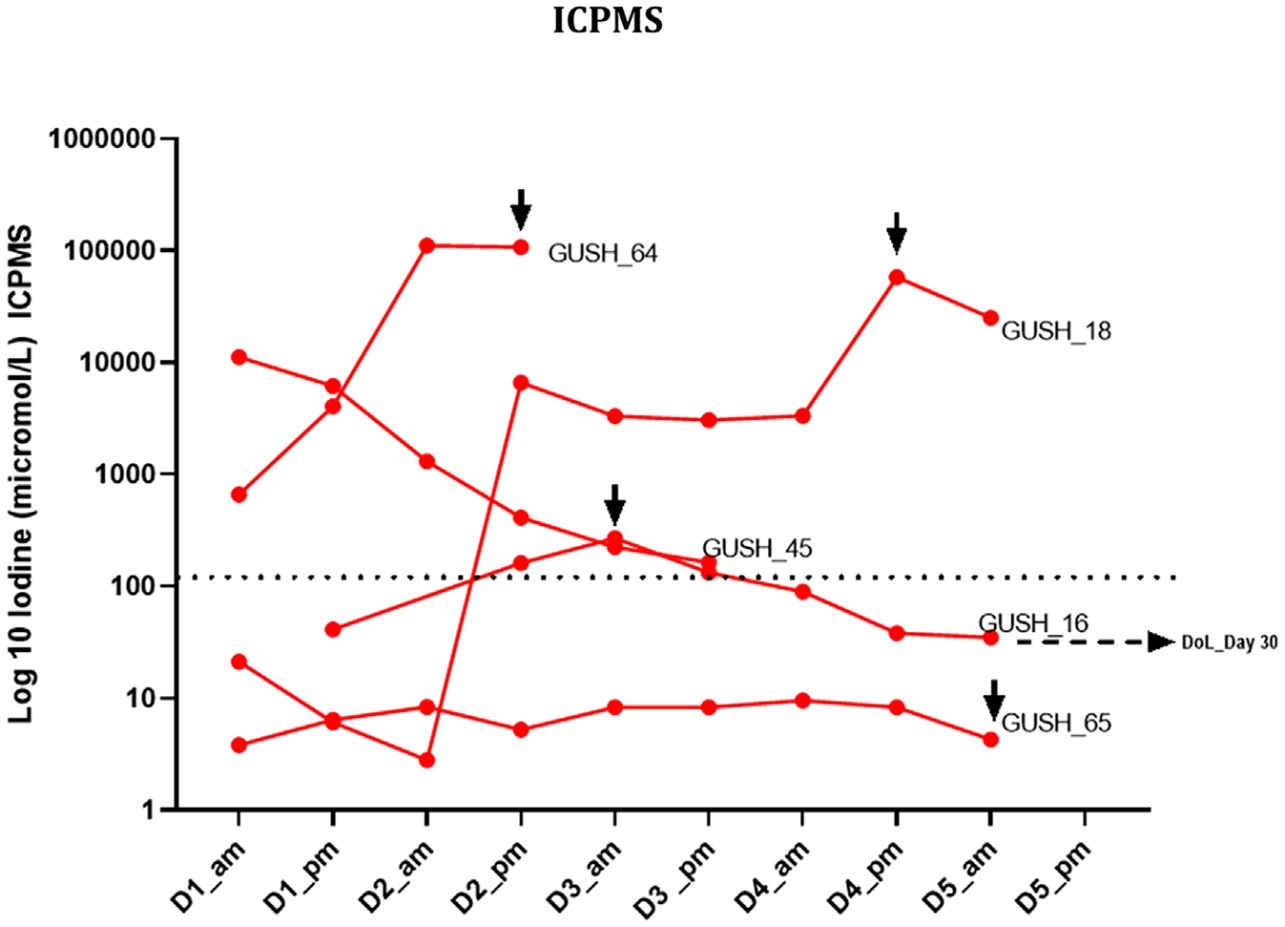
Time course of drain fluid iodine in patients experiencing anastomotic leak (AL) as measured by inductively coupled plasma mass spectroscopy (ICPMS) (D1 etc., day 1 etc.). Arrows indicate the day of clinical diagnosis of AL; the dotted line represents the cutoff derived from the highest mean at any day plus three standard deviations in the non‐AL group (cutoff, background, was 130 μmol/L, D1_am).

**FIGURE 4:**
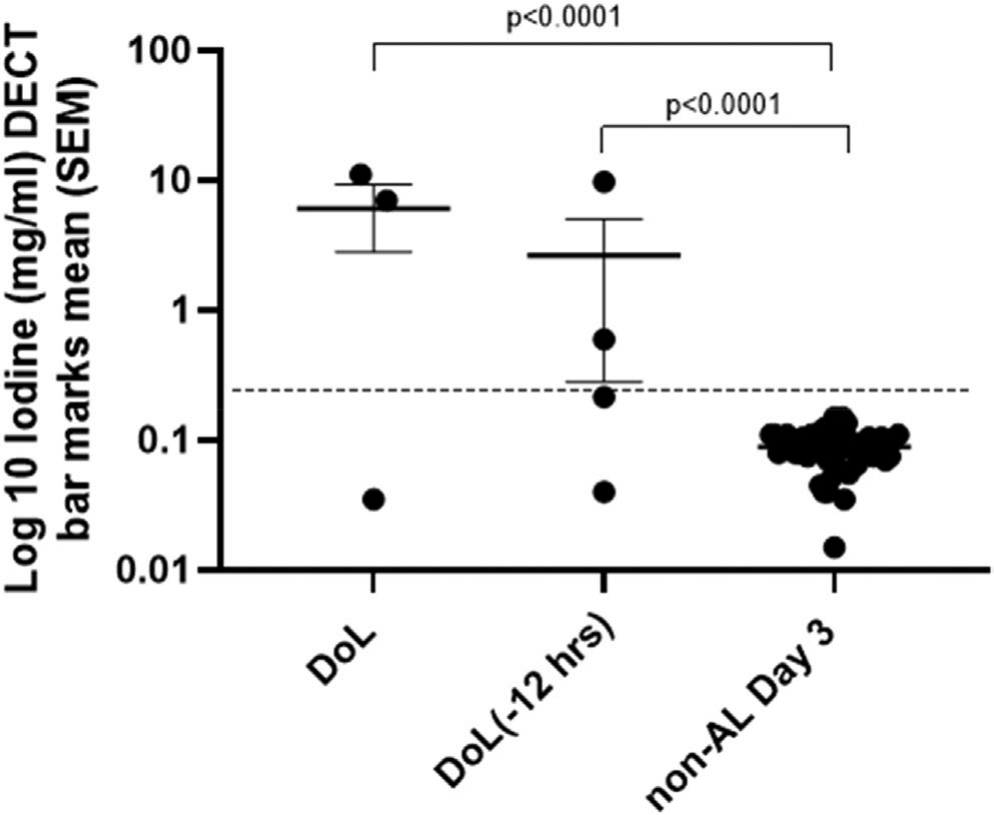
Drain fluid iodine on the day of anastomotic leak (AL) versus day 3 of non‐AL (dual‐energy computed tomography, DECT) and day 3 (−12 h). DoL, day of anastomotic leak; DoL (−12 h), 12 h before diagnosed day of leak. The dotted line marks the cutoff (background mean of the non‐AL group plus three standard deviations).

**FIGURE 5:**
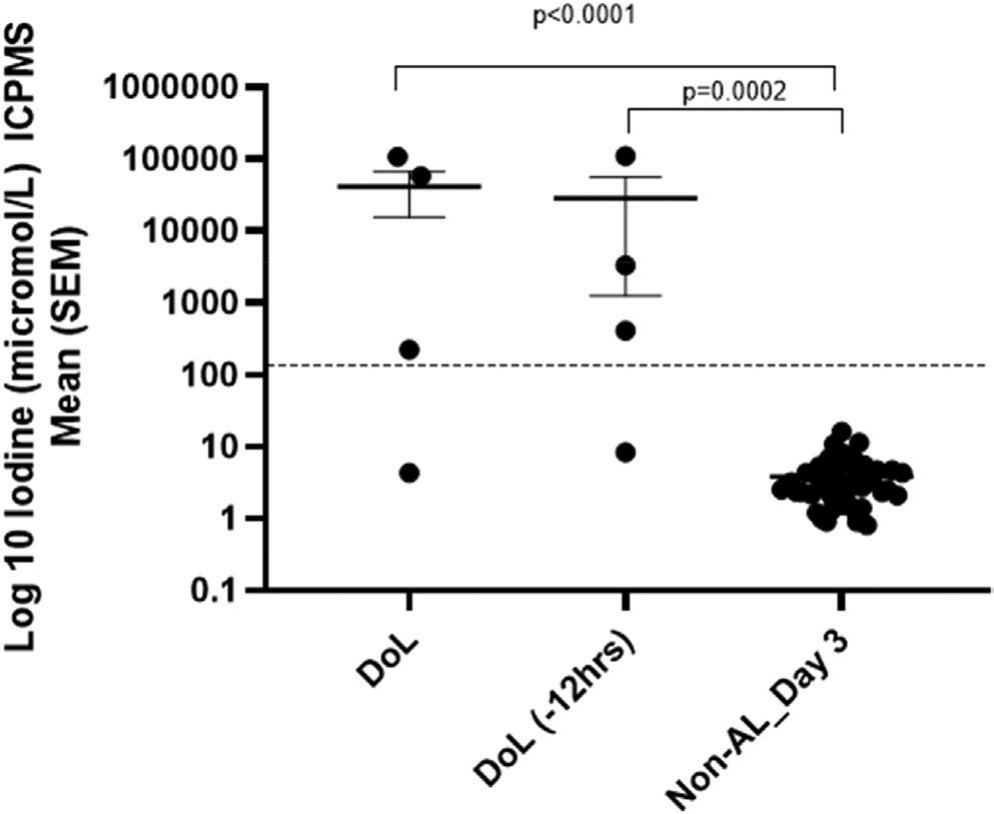
Drain fluid iodine on day of anastomotic leak (AL) versus day 3 of non‐AL (induction coupled mass spectroscopy, ICPMS). DoL, day of anastomotic leak. The dotted line marks the cutoff (background mean of the non‐AL group plus three standard deviations).

**FIGURE 6:**
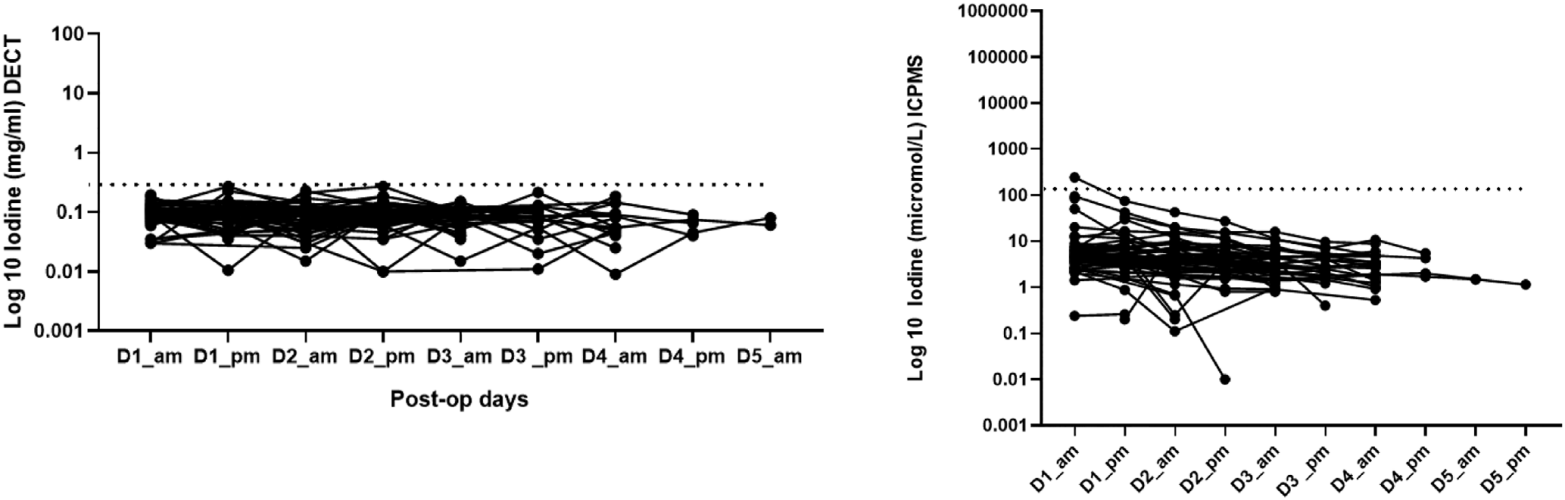
Iodine measured in the nonanastomotic leak (non‐AL) group by dual‐energy computed tomography (DECT) and inductively coupled plasma mass spectroscopy (ICPMS). (A) DECT‐measured iodine levels in the non‐AL group. (B) ICPMS measured iodine in the non‐AL group. D1, postoperative day 1. The dotted line marks the cutoff (background mean of the non‐AL group plus three standard deviations).

A cutoff value for diagnosis of AL was derived by taking the highest value of the daily mean plus three standard deviations of the patients who did not experience an AL (Figures 2, 3, 4, 5, 6). In all these figures this cutoff is represented as a dotted line.

The two methodologies for the detection of iodine in GG (DECT and ICPMS) are depicted in Figures 2 and 3 and the *y*‐axis is presented as a logarithmic scale due to the extremely high value range. [Note: the unit of measurement of iodine in GG was mg/mL when measured by DECT and μmol/L for ICPMS. For one patient, GUSH_65, there was insufficient sample volume to permit a scan for DECT, but enough for ICPMS. The patient who experienced the delayed AL (at postoperative day 30) did not have any specimens for analysis as the drain had been removed.]

When evaluated by DECT, the patients experiencing an AL had an elevated drain fluid iodine compared with those patients who did not. Mean iodine levels on the day of AL (DoL) were 6.05 mg/mL vs. 0.088 mg/mL in the non‐AL group (*p* < 0.0001) and iodine levels 12 h before the DoL were also significantly higher (2.65 mg/mL) than background (0.088 mg/mL, *p* < 0.0001) (Figure 4).

When evaluated by ICPMS, the patients experiencing AL had a significantly elevated mean drain fluid iodine compared with those who did not (mean DoL 41 437 μmol/L vs. non‐AL 3.81 μmol/L, *p* < 0.0001) and iodine levels in drain fluid 12 h before diagnosed AL were 28 601 μmol/L vs. 3.81 μmol/L in the non‐AL group (*p* = 0.0002) (Figure 5).

### Rectal tube iodine

Samples collected from the rectal tube 30 min after administration of GG acted as a positive control and to quantify the intraluminal levels. These data show the gradient across the intraluminal to extraluminal space. When rectal tube specimens were measured by DECT, the measurements peaked at a maximum of 37.71 mg iodine/mL (±SD 7.064). This was previously shown to be the maximum level measurable by DECT [12].

When rectal tube specimens were measured by ICPMS, the measured iodine in stool was 1 803 240 μmol/L (SD ±813 987 μmol/L). This was only studied in four specimens as the required dilutions were considerable. The mean intraperitoneal level of iodine in the non‐AL patients was 3.81 μmol/L.

No adverse effects were observed or attributable to the GG flushes per se.

### Long‐term follow‐up

At long‐term postsurgical follow‐up, all patients who experienced an AL during the study had their ileostomy closed and intestinal continuity restored. The one patient who the grade B AL and was managed with image‐guided drainage did not require any further intervention.

### Cutoff values

A cutoff value for diagnosis of AL was derived by taking the highest value of the daily mean plus three standard deviations of the patients who did not experience an AL. For DECT the cutoff was 0.240 mg iodine/mL in non‐AL drain fluid, observed as highest on day 1 pm. The cutoff for ICPMS was 130 μmol/L, observed on day 1 am. (Figure 6).

## DISCUSSION

This prospective, observational, 2b exploration cohort study showed that drain fluid iodine can be used as a biomarker for the detection of AL when the rectal tube is flushed with GG and a diverting ileostomy is omitted.

The two methods of iodine measurement (DECT and ICPMS) can be performed on an early morning drain fluid specimen and have timely results available to inform clinical practice, with the goal of intervention before serious clinical deterioration [11].

ICPMS was able to detect the AL in all four patients who had their pelvic drain situated in the pelvis. Indeed, the mean drain fluid iodine level in patients who experienced an AL was over 10 000 times higher than in those who did not experience an AL. ICPMS also showed a signal, rising above the defined cutoff, for a delayed AL that was maximum at postoperative day 3 and was subsequently diagnosed as a pelvic abscess at postoperative day 30. The one patient who had a malpositioned drain, understandably, did not see a rise in drain fluid iodine as measured by ICPMS or DECT. This false negative is an important limitation of all drain fluid BM studies.

The iodine measurements of drain fluid collected 12 h prior to the clinical detection and diagnosis of the AL were also high and statistically significantly elevated compared with the patients who did not experience an AL. This may prove to be very helpful information and an adjunct to the early diagnosis of AL before clinical deterioration. One patient initially had elevated drain fluid iodine (Figure 2) but it had returned to below the derived threshold on the day of clinical diagnosis. A second patient had an additional drain fluid iodine elevation well before clinical diagnosis, but this was only detected on ICPMS (Figure 3). These drain fluid results may have led to an earlier diagnosis if they were available to the treating clinicians.

This study also aimed to define clinically relevant cutoff values for the two methodologies. These are presented in Figures 2 and 3 as dotted lines, and may be used to aid the clinician with an instantaneous ‘value of concern’ rather than looking for a trend over time. This was made possible due to the nature of EILS and the fact that the mean drain fluid iodine measured by DECT (AL versus non‐AL) was almost two orders of magnitude higher, and over four orders of magnitude higher when ICPMS was employed. Figure 6 shows the derived cutoff value against all the non‐AL patients in a time plot. The cutoff value was defined as the highest mean plus three standard deviations observed during the time course.

The use of GG as a rectal tube patency flush is a novel concept and may have other benefits than just the delivery of an iodine‐rich fluid to the intestinal lumen. Thirty milliliters of GG was delivered four times a day and there were no adverse events related to these flushes. The median day on which full diet was first tolerated was day 1 (Table 1). The patients were managed with an early enteral feeding protocol and the overall length of stay of 5 days compares favourably with that reported in a systematic review of 17 randomized controlled trials that found a median length of stay of between 4 and 16 days when an early feeding programme was studied [23]. Perhaps GG flushes of the left colon may speed gastrointestinal recovery.

Many drain fluid BMs have been studied. Most of these are predictive and few are diagnostic of AL [24], Drain fluid cytokine BMs look at the environment around the anastomosis and don't distinguish between the inherent inflammation of healing and remodelling, and the individual's unique response to that process. EILS make intuitive sense as a research target as they would closely mimic the process of extravasation and AL [1].

The day 3 C‐reactive protein was significantly higher in those patients who experienced an AL. This systemic BM measures inflammation and repair occurring in the individual and is less specific for AL than EILS [24].

In other studies from this institution, extravasation of the enzyme amylase was measured in the drain fluid of patients undergoing restorative surgery with an ileal J pouch for ulcerative colitis and without a diverting loop ileostomy [7]. That study found very high levels of the enzyme within the lumen of the pouch (over 1000 times the serum reference range) but levels in the drain fluid of those patients who did not experience an AL approximated the serum levels. The peritoneal levels were essentially an ultrafiltrate of the serum levels. That study concluded that this extravasated intraluminal enzyme rose significantly in drain fluid samples of those patients who did experience an AL. Importantly, an elevation in these levels was detected on the day prior to clinical deterioration. This led to the conclusion that drain fluid analysis of EILS may allow for the early detection of AL before clinical deterioration of the patient. Amylase is more likely to be sensitive after ileal pouch surgery in view of the more proximal anatomical location of the anastomosis in the gastrointestinal tract compared with a colorectal anastomosis [8]. The studies presented here sought to define a cutoff level above which a ‘positive’ finding can be made to permit an ‘instantaneous’ abnormal finding [7, 8].

A further study evaluating drain fluid amylase (DFA) in colorectal anastomoses showed a statistically significant elevation in DFA in patients who experienced AL [9]. It was found that 28 of 61 patients did not have measurable levels of amylase in the rectal tube by postoperative day 2 and hence false negatives in those patients would be predicted had they experienced an AL. The investigation of drain fluid iodine after rectal tube flushes with GG aimed to overcome this limitation by ensuring that the BM was in high concentrations in the lumen of the neorectum.

A limitation of this study is the absence of a ‘no test’ control group. This was an observational study and the drain fluid measurements were not available to the treating clinicians. There have been no drain fluid BM studies in colorectal surgery in which the results have been available to the clinicians [1]. That information would be most valuably studied in a randomized controlled trial of GG flushes with unblinded ‘test’ and ‘no test’ arms. GG flushes may see a reduction in ileus and a quicker return to diet, as is implied in these data, but that would have to be tested in a randomized study of GG flushes versus saline flushes. As all eligible patients did not have a DI, these patients represent a cohort at a predicted lower risk of AL. This study did not assess the parameters considered by the surgeons to arrive at the decision not to defunction the colorectal anastomoses.

This study did not evaluate the patient experience and patient‐reported outcome measures could assess the rectal tubes and the patient preference for avoiding a stoma [25]. A cost‐effectiveness study would also be helpful to evaluate the intervention but would need to balance the savings of avoiding a second operation in those patients who avoided a stoma and their increased productivity with fewer days away from work.

The aim of this exploration study was to evaluate the efficacy of the measurement of drain fluid iodine by DECT and ICPMS and associate the results with the occurrence of AL.

## CONCLUSIONS

In the end, the decision to omit a DI is a balance of avoiding harm to the majority of patients who do not leak and mitigating the septic consequences to the minority that ultimately do so. This decision is framed by the understanding that early return to theatre and diversion may see similar outcomes for those patients who are going to experience AL compared with those who are diverted at the index resection. Drain fluid BM may aid in the early detection and remediation of AL before clinical deterioration occurs.

## AUTHOR CONTRIBUTIONS

**David A. Clark:** Conceptualization; investigation; funding acquisition; writing – original draft; methodology; writing – review and editing; project administration; supervision. **Karen Dobeli:** Methodology; validation; writing – review and editing; data curation; investigation; formal analysis. **Darren Allen:** Investigation; writing – review and editing; validation; methodology; data curation; formal analysis. **Brett McWhinney:** Investigation; methodology; validation; writing – review and editing; formal analysis; data curation. **Michael Lonne:** Methodology; writing – review and editing; formal analysis. **Aleksandra Edmundson:** Conceptualization; investigation; methodology; validation; visualization; writing – review and editing; formal analysis; project administration; data curation.

## FUNDING INFORMATION

This study was supported by the Wesley Research Institute through the award of a competitive research project grant of AUD$28 280.

## CONFLICT OF INTEREST STATEMENT

The authors have no conflict of interest to declare.

## ETHICS STATEMENT

Ethics approval was granted by the Royal Brisbane and Women's Hospital Human Research and Ethics Committee (HREC/2020/QRBW/64118), the Wesley Hospital Human Research and Ethics Committee (2020.17.327) and the St Vincent's Health and Aged Care Human Research Ethics Committee (HREC 20/10).

## DATA AVAILABILTY STATEMENT

Supporting data are available on request.

## PATIENT CONSENT

Signed patient information consent forms were retained for all participants in the study.

## TRIAL REGISTRATION

Trial registration with the Australian New Zealand Clinical Trials Registry (ACTRN12620000710921).

## Supporting information


**Supplementary 1**.

## References

[1] Clark DA , Steffens D , Solomon M . An umbrella systematic review of drain fluid analysis in colorectal surgery for the detection of anastomotic leak: not yet ready to translate research studies into clinical practice. Colorectal Dis. 2021;23:2795–2805. 34314559 10.1111/codi.15844

[2] Clark DA , Stephensen B , Edmundson A , Steffens D , Solomon M . Geographical variation in the use of diverting loop ileostomy in Australia and New Zealand colorectal surgeons. Ann Coloproctol. 2021;37:337–345. 32972099 10.3393/ac.2020.09.14.1 PMC8566141

[3] McGiffin T , Clark DA , Edmundson A , Steffens D , Stevenson A , Solomon M . Surgical management and long‐term functional outcomes after anastomotic leak in patients undergoing minimally invasive restorative rectal resection and without a diverting ileostomy. ANZ J Surg. 2022;92:806–812. 35072326 10.1111/ans.17475

[4] Boyce SA , Harris C , Stevenson A , Lumley J , Clark D . Management of low colorectal anastomotic leakage in the laparoscopic era: more than a decade of experience. Dis Colon Rectum. 2017;60:807–814. 28682966 10.1097/DCR.0000000000000822

[5] Clark DA , Stevenson A , Lumley J , Petersen D , Harris C , Steffens D , et al. Does an ileostomy cover the surgeon or the anastomosis? ANZ J Surg. 2022;92:19–20. 35212109 10.1111/ans.17364

[6] Singh PP , Zeng ISL , Srinivasa S , Lemanu DP , Connolly AB , Hill AG . Systematic review and meta‐analysis of use of serum C‐reactive protein levels to predict anastomotic leak after colorectal surgery: use of C‐reactive protein levels to predict anastomotic leak after colorectal surgery. Br J Surg. 2014;101:339–346. 24311257 10.1002/bjs.9354

[7] Clark DA , Cuda T , Riddell A , Radford‐Smith G , Solomon M . Drain fluid amylase as a sensitive biomarker for the early detection of anastomotic leakage in ileal pouch surgery. Colorectal Dis. 2019;21:460–464. 30565365 10.1111/codi.14536

[8] Clark DA , Edmundson A , Steffens D , Radford‐Smith G , Solomon M . Multicenter study of drain fluid amylase as a biomarker for the detection of anastomotic leakage after Ileal pouch surgery without a diverting ileostomy. Dis Colon Rectum. 2022;65:1335–1341. 35358101 10.1097/DCR.0000000000002376

[9] Clark DA , Edmundson A , Steffens D , Harris C , Stevenson A , Solomon M . Drain fluid amylase as a biomarker for the detection of anastomotic leakage after rectal resection without a diverting ileostomy. ANZ J Surg. 2022;92:813–818. 34994080 10.1111/ans.17461

[10] Clark DA , Cuda T , Pretorius C , Edmundson A , Solomon M , Riddell AD . Amylase quantification in the terminal ileum following formation of an ileostomy. Sci Rep. 2020;10:19368. 33168838 10.1038/s41598-020-76349-y PMC7652869

[11] Clark DA , Yeoh E , Edmundson A , Harris C , Stevenson A , Steffens D , et al. A development study of drain fluid gastrografin as a biomarker of anastomotic leak. Ann Coloproctol. 2022;38:124–132. 33445840 10.3393/ac.2020.12.24 PMC9021859

[12] Clark DA , Yeoh E , Edmundson A , Pratap J , Snow T , Solomon M , et al. Gastrografin can be detected in ex vivo biological specimens by dual‐energy CT scanning. J Med Imaging Radiat Oncol. 2020;64:634–640. 32543123 10.1111/1754-9485.13071

[13] Xiao L , Zhang W‐b , Jiang P‐c , Bu X‐f , Yan Q , Li H , et al. Can Transanal tube placement after anterior resection for rectal carcinoma reduce anastomotic leakage rate? A single‐institution prospective randomized study. World J Surg. 2011;35:1367–1377. 21437746 10.1007/s00268-011-1053-3

[14] Shigeta K , Okabayashi K , Baba H , Hasegawa H , Tsuruta M , Yamafuji K , et al. A meta‐analysis of the use of a transanal drainage tube to prevent anastomotic leakage after anterior resection by double‐stapling technique for rectal cancer. Surg Endosc. 2016;30:543–550. 26091985 10.1007/s00464-015-4237-3

[15] Dindo D , Demartines N , Clavien P‐A . Classification of surgical complications: a new proposal with evaluation in a cohort of 6336 patients and results of a survey. Ann Surg. 2004;240:205–213. 15273542 10.1097/01.sla.0000133083.54934.ae PMC1360123

[16] Rahbari NN , Weitz J , Hohenberger W , Heald RJ , Moran B , Ulrich A , et al. Definition and grading of anastomotic leakage following anterior resection of the rectum: a proposal by the International Study Group of Rectal Cancer. Surgery. 2010;147:339–351. 20004450 10.1016/j.surg.2009.10.012

[17] Flores‐Funes D , Campillo‐Soto Á , Pellicer‐Franco E , Aguayo‐Albasini JL . The use of coffee, chewing‐gum and gastrograffin in the management of postoperative ileus: a review of current evidence. Cir Esp. 2016;94:495–501. 27456544 10.1016/j.ciresp.2016.05.020

[18] Habib K , Gupta A , White D , Mazari FAK , Wilson TR . Utility of contrast enema to assess anastomotic integrity and the natural history of radiological leaks after low rectal surgery: systematic review and meta‐analysis. Int J Colorectal Dis. 2015;30:1007–1014. 25922145 10.1007/s00384-015-2225-7

[19] Chintamani RB . Randomized clinical study of Gastrografin administration in patients with adhesive small bowel obstruction (Br J Surg 2003; 90: 542–546). Br J Surg. 2003;90:1165. 10.1002/bjs.4150 12734858

[20] Biondo S , Miquel J , Espin‐Basany E , Sanchez JL , Golda T , Ferrer‐Artola AM , et al. A double‐blinded randomized clinical study on the therapeutic effect of Gastrografin in prolonged postoperative ileus after elective colorectal surgery. World J Surg. 2016;40:206–214. 26446450 10.1007/s00268-015-3260-9

[21] Aran S , Shaqdan KW , Abujudeh HH . Dual‐energy computed tomography (DECT) in emergency radiology: basic principles, techniques, and limitations. Emerg Radiol. 2014;21:391–405. 24676736 10.1007/s10140-014-1208-2

[22] Wilschefski SC , Baxter MR . Inductively coupled plasma mass spectrometry: introduction to analytical aspects. Clin Biochem Rev. 2019;40:115–133. 31530963 10.33176/AACB-19-00024 PMC6719745

[23] Herbert G , Perry R , Andersen HK , Atkinson C , Penfold C , Lewis SJ , et al. Early enteral nutrition within 24 hours of lower gastrointestinal surgery versus later commencement for length of hospital stay and postoperative complications. Cochrane Database Syst Rev. 2019;7:Cd004080. 31329285 10.1002/14651858.CD004080.pub4 PMC6645186

[24] Su'a BU , Mikaere HL , Rahiri JL , Bissett IB , Hill AG . Systematic review of the role of biomarkers in diagnosing anastomotic leakage following colorectal surgery. Br J Surg. 2017;104:503–512. 28295255 10.1002/bjs.10487

[25] Mackay I , Clark DA , Nicholson J , Edmundson A , Steffens D , Solomon M . Risk taking propensity: nurse, surgeon and patient preferences for diverting ileostomy. Colorectal Dis. 2022;24:1073–1079. 35426482 10.1111/codi.16149 PMC9790330

