# Supplementary material for: Drain fluid iodine as a biomarker of anastomotic leak after low anterior resection in patients undergoing Gastrografin rectal tube flushes and omission of a diverting ileostomy: The GUSH study
Source: Colorectal Dis. 2025 Feb 19;27(2):e70031. doi: 10.1111/codi.70031 (PMC11840294; doi:10.1111/codi.70031)
Supplement: Supplementary file 1 — Supplementary 1. [file CODI-27-0-s001.docx]

**Supplement 1.**

**Iodine measurement by DECT**

The application of dual-energy CT scanning in the measurement of iodine in clinical specimens was established in a prior study from this institution. [1] DECT, also known as spectral CT, is a new technology that allows simultaneous or near-simultaneous acquisition of two datasets from the same anatomical region at different x-ray tube voltages. [2] The x-ray absorption properties of materials are dependent on the energy of the incident x-ray photons, and the relative change in x-ray absorption at different photon energies varies between materials. Hence, DECT is capable of differentiating materials with different atomic numbers. The organically bound iodine in Gastrografin is able to be simply quantitated using a DECT protocol described in the literature. [3] The lower limit of detection of a solution containing GG was at a concentration of 0.097%, which correlated to an iodine density of >1 mg/ml. The upper limit of the true negative (or background) levels of iodine measured in drain fluid by DECT was derived by calculating the mean (+ 3 SD) iodine density (mg/ml) in GG negative samples. The phase 1 study established measurements of iodine density above 1.2 mg/ml to represent a positive result for the presence of GG in a solution measured by DECT. [1] The present study recalculated this cut-off with a larger sample size and was performed by the same radiographer (KD) using the same DECT scanner for all drain fluid samples.

DECT data were obtained using a dual detector layer spectral CT (IQon Elite, Philips Healthcare, The Netherlands). All samples for individual participants were placed sequentially along the z-axis on the CT couch in the isocentre of the gantry and imaged in a single acquisition. The same three (3) control samples were included in the scanned field with each CT acquisition. CT data were acquired with tube voltage of 120 kVp, tube current-time product of 100 mAs, pitch 1.091, detector collimation 32 x 0.625, CT volume dose index (CTDI_vol_) 7.5 mGy.

The DECT datasets were loaded into a clinical spectral CT analysis software (IntelliSpace Portal V11.1, Philips Healthcare, The Netherlands). A region of interest (ROI) was placed within the collected fluid for each sample on the ‘Iodine Density’ spectral reconstruction and the average density of iodine, in grams/millilitre, contained within the ROIs was recorded. The size and position of the ROIs varied for each sample and were aimed to include a substantial sample of the fluid while avoiding partial volume effect.

References

1. Clark DA, Yeoh E, Edmundson A, Harris C, Stevenson A, Steffens D, et al. A development study of drain fluid gastrografin as a biomarker of anastomotic leak. *Ann Coloproctol* 2022; **38:** 124-32.

2. Aran S, Shaqdan KW, Abujudeh HH. Dual-energy computed tomography (DECT) in emergency radiology: basic principles, techniques, and limitations. *Emerg Radiol* 2014; **21:** 391-405.

3. Clark DA, Yeoh E, Edmundson A, Pratap J, Snow T, Solomon M, et al. Gastrografin can be detected in ex vivo biological specimens by dual-energy CT scanning. *J Med Imaging Radiat Oncol* 2020; **64:** 634-40.
